# Supplementary material for: Rapid multiple-quantum three-dimensional fluorescence spectroscopy disentangles quantum pathways
Source: Nat Commun. 2019 Oct 18;10:4735. doi: 10.1038/s41467-019-12602-x (PMC6800439; doi:10.1038/s41467-019-12602-x)
Supplement: Supplementary file 1 — Supplementary Information [file 41467_2019_12602_MOESM1_ESM.pdf]

Supplementary Information for

"Rapid multiple-quantum three-dimensional fluorescence spectroscopy disentangles quantum pathways"

Mueller et al.

Supplementary Note 1: Details of the Experiment

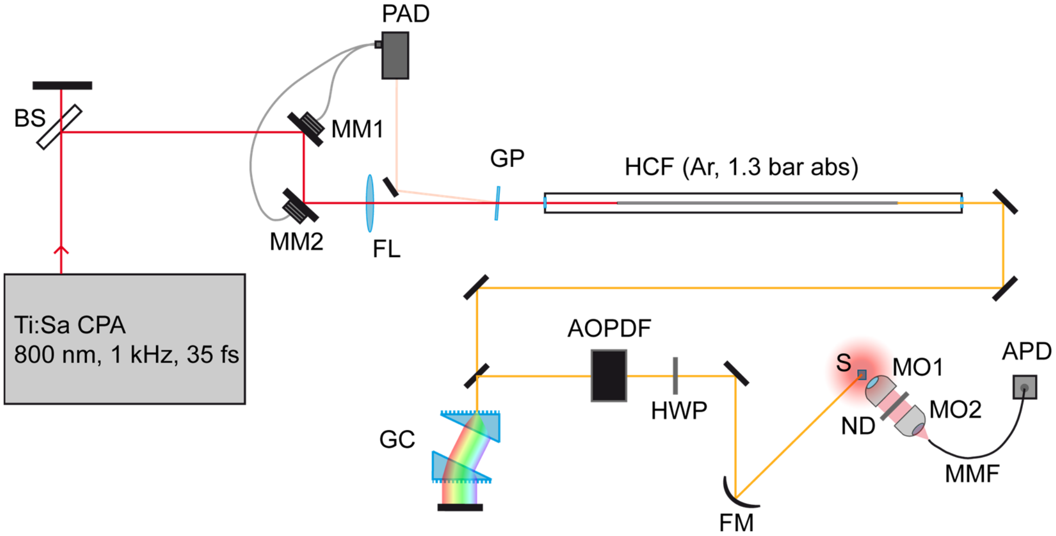

Supplementary Figure 1. Schematic of shot-to-shot single-beam multidimensional fluorescence spectroscopy. CPA: chirped-pulse amplifier; BS: beam splitter; MM1, MM2: motorized mirrors; FL: fused-silica focusing lens ($f = 120$ cm); PAD: position and angle detector; GP: fused-silica glass plate; HCF: hollow-core fiber (filled with argon); GC: dual grism compressor; AOPDF: acousto-optic programmable dispersive filter; HWP: half-wave plate; FM: focusing mirror ($f = -15$ cm); S: sample, being pumped through a capillary flow cuvette; MO1, MO2: microscope objectives, ND: neutral density filter; MMF: multimode fiber; APD: avalanche photodiode.

The schematic layout of our experimental apparatus is shown in Supplementary Figure 1. We guide a fraction (0.4 mJ) of the 35 fs pulses (as confirmed by intensity autocorrelation) of a commercial Ti:Sa chirped-pulse amplifier laser system (Spitfire Pro, Spectra Physics) with 1 kHz repetition rate through a beam-stabilization system (Aligna, TEM Messtechnik GmbH). The latter consists of two motorized mirrors which are controlled via a feedback from a combined angle and position detector. This detector tracks the spatial properties of a weak reflection of the fundamental beam that is produced by a fused-silica glass plate and led into the detection unit. With that, a stabilized beam pointing into the argon-filled fused-silica hollow-core fiber with a length of 110 cm and an inner diameter of 250 μm (HCF, Ultrafast Innovations GmbH) via a lens with $f = 120$ cm is ensured, providing long-term stability of the fiber output. The argon pressure inside the fiber is kept constant at 1.3 bar (absolute). Dispersion management is provided by a dual grism compressor (Fastlite), especially in order to pre-compensate the dispersion which is introduced by the 2.5 cm long TeO_2 crystal of the pulse shaper. The pulse shaper is an acousto-optic programmable dispersive filter (AOPDF, DAZZLER, Fastlite) which operates directly at the repetition rate of the laser on a shot-to-shot basis. With that, we generate collinear four-pulse trains which are then focused via a focusing mirror ($f = -15$ cm) into the sample. The sample is pumped through a capillary-type flow cell with square cross section (250 μm)^2 (131.130-QS, Hellma), which is placed vertically into the focus position. We use a peristaltic pump (Masterflex L/S model 7518-00). The tubing circuit is connected to a reservoir with ~25 mL sample volume, where the concentration of 6,13-bis((triisopropylsilyl)ethynyl)quinoxalino[2,3-b]phenazine (TIPS-TAP^{2-}) in THF is 0.4 mM. Filling the reservoir and sealing the tubing circuit is conducted inside an argon-filled glove box. The excitation energy is 200 nJ at maximum temporal overlap and complete interference of all four excitation pulses. At an angle of 90° with respect to the excitation direction, two microscope objectives with 0.25-NA (04OAS010, CVI MellesGriot) collect the fluorescence light which is then guided into a 0.22-NA multimode glass fiber (QP400-2-SR, Ocean Optics, core diameter 400 μm). The detection of stray light is efficiently avoided by utilizing the 90° detection geometry in combination with a horizontal excitation beam polarization which is generated via a half-wave plate after the AOPDF. For the detection of fluorescence spectra and confirmation of the absence of any stray light (see also Supplementary Figure 3), we plug the fiber into a spectrometer (HR 4000, Ocean Optics, not shown in Supplementary

Figure 1). In order to perform multidimensional experiments, the fiber is plugged into an avalanche photodiode (APD410x, Thorlabs). The fluorescence is attenuated by an absorptive neutral density filter (FS-3R, Newport) with OD = 1.8 to a suitable signal level to avoid artifacts stemming from saturation of the APD. The analog fluorescence signal is finally digitized by a 14 bit 1 GS/s digitizer card (ADQ14, Signal Processing Devices Sweden AB). The data presented in the manuscript were averaged over four complete datasets. In Supplementary Figure 2, we show exemplarily the fourth-order 1Q-0Q-1Q and the sixth-order 2Q-0Q-1Q 3D spectra resulting from a single data set without averaging. The same plotting parameters were used as those described in the captions of manuscript Figs. 3 and 5, respectively.

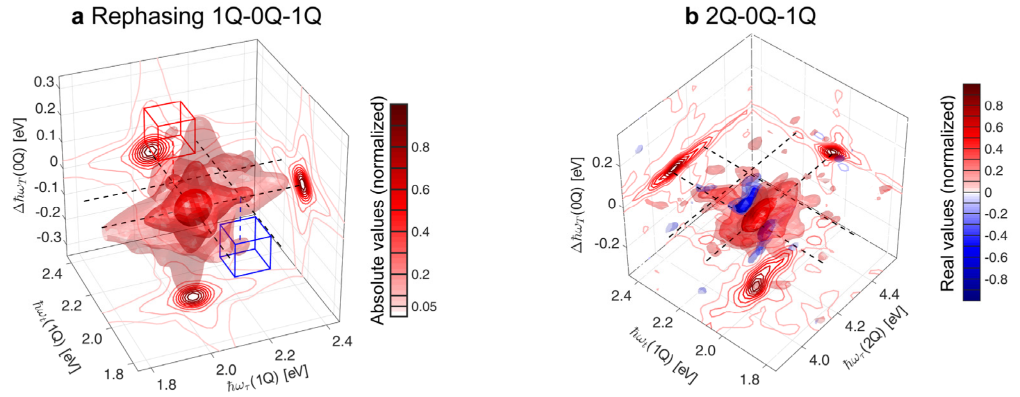

Supplementary Figure 2: 3D spectra obtained after a single measurement without averaging. **a** Rephasing 1Q-0Q-1Q and **b** 2Q-0Q-1Q 3D spectra, generated by the sum of real-valued rephasing and nonrephasing 2Q-0Q-1Q contributions.

It is evident that an excellent signal-to-noise ratio is achieved for the fourth-order 3D spectrum after only a single run, i.e., without any averaging. For the sixth-order contribution that is much weaker, the noise is significantly higher compared to the four-times averaged data in Fig. 5 of the manuscript. Nevertheless, relevant signal features are already visible. This shows that it is indeed possible to obtain all fourth-order signals in 8 min, while for the sixth-order signals additional averaging is helpful.

We want to turn now to a discussion regarding the sample stability and the effect of its degradation on the acquired data. As a dianionic compound, TIPS-TAP^{2-} is extremely sensitive towards even traces of oxygen and moisture. Although care was taken during preparation, i.e., flame-drying of the sample reservoir flask and assembly of the tubing circuit inside the glove box, it was not possible to achieve stability of the compound within the tubing circuit over several hours. We believe that either residual traces of oxygen and moisture inside the tubing circuit or an insufficient sealing of the latter caused the sample decay. Fluorescence spectra of TIPS-TAP^{2-} in THF, taken immediately before and after the multidimensional experiment and normalized to the maximal value of the former are presented in Supplementary Figure 3. Both spectra are acquired by using the same excitation energy. The fluorescence peaks at 603 nm and 656 nm feature a well-resolved vibrational progression. We do not observe any scattered light of the excitation spectrum which spans the region between 550 and 635 nm, peaking at 590 nm, nor any additional fluorescence bands of possible decay products. Moreover, we do not observe any additional peaks that do not belong to the dianionic species in all three-dimensional (3D) spectra. Thus, we conclude that we measured the nonlinear response of TIPS-TAP^{2-} selectively in our 3D experiment. We note further that fluorescence detection here selectively captures the nonlinear signals from the highly-fluorescent dianion only, while in an approach using coherence detection, one cannot exclude signals from non-fluorescent decay products.

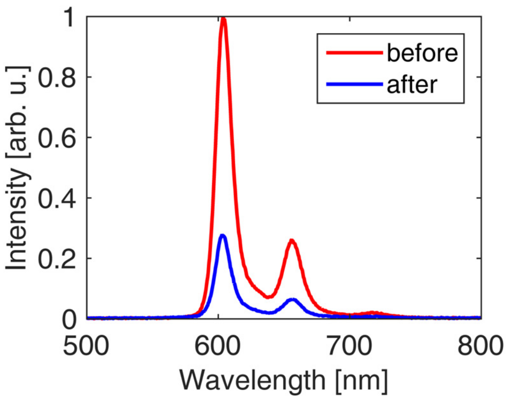

Supplementary Figure 3. Fluorescence spectra of TIPS-TAP^{2-} in THF. The spectra shown are measured immediately before (red) and after (blue) the three-dimensional experiment with the same excitation power and normalized to the maximal value of the red curve.

The question arises if the sample degradation would affect the reconstruction of nonlinear signals. We will now discuss why our sampling scheme avoids to first order those errors that might be connected with sample decay.

The most crucial criterion for extracting nonlinear contributions according to their phase signatures by Eq. (1) in the manuscript is that the signal level should not drop dramatically within the phase-cycling procedure for any particular given setting of time delays. Hence, it would be detrimental if we had sampled the phase-cycling steps after scanning the complete manifold of interpulse time delays. In our sampling scheme, however, we first acquire all the phase-cycling steps for any given combination of interpulse delays before we change the latter to different settings. Due to the availability of shot-to-shot pulse-shape modulation, acquiring a 125-step phase-cycling set takes 125 ms at 1 kHz repetition rate. This time scale is significantly smaller than the time scale on which the sample degrades, so that the phase-cycling procedure is not compromised by sample degradation.

We characterize the sample degradation during the measurement by recording several “reference” data points, consisting of a repetition (for averaging) of a single, compressed, but otherwise unshaped laser pulse. This reference measurement corresponds effectively to setting all interpulse time delays and interpulse phase differences to zero. These reference data are acquired at the beginning and the end of the full dataset as well as before any population time increment. Thus, between two averaged reference data measurements, the 125-step phase cycling is conducted for all delay increments of τ and t at a given, fixed T. The resulting reference data points are shown in Supplementary Figure 4.

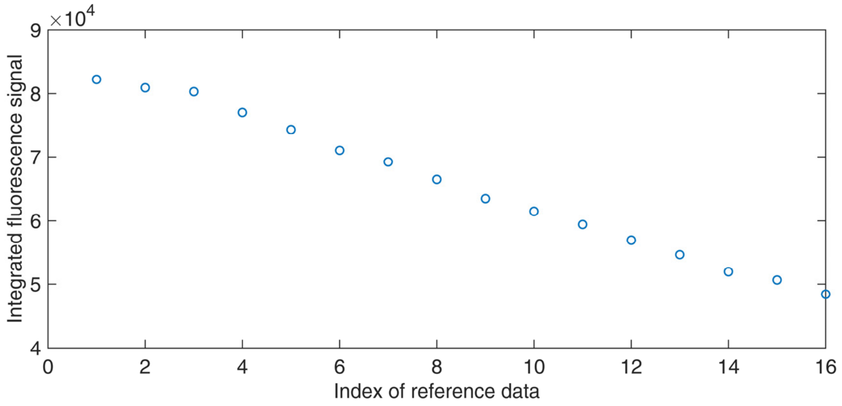

Supplementary Figure 4. Reference measurements. The plot shows time-integrated fluorescence signals that were acquired as reference data (by averaging over a repetition of a single laser pulse) during the multidimensional experiment. Between any two reference points, 125 different phase-cycled data points for each τ and t delay are acquired (not shown).

In our specific case, analyzing the decay of the reference measurements, we find that the signal changes on average by 3.5% between two reference measurements, so that the claim of constant intensity for any given phase-cycling set is fulfilled up to that precision throughout all τ and t combinations for any T, and the intensity decreases by less than 0.02% for a 125-step phase cycling protocol at any fixed τ and t combination.

The signal decays for instable samples throughout the sampling of T steps because these are sampled last. The degradation acts then as an additional damping of the actual dephasing dynamics of coherences that evolve during T. This has most effects on the 0Q coherence dynamics which usually dephase within several picoseconds. The sample decay then manifests as an additional broadening of the 3D line shapes along the ω_T axis in the 3D spectra. However, a broadening along ω_T for 0Q coherences is introduced anyway by the Hann window we applied in order to avoid truncation artifacts from Fourier transform. Hence, this additional broadening by sample decay might be present but it does not change the spectral position of 0Q features.

Finally, we point out that a sample decay over the course of the acquisition time is not particular in any way to the present technique, but would rather occur with any other approach. On the contrary, using the present shot-to-shot modulation technique, the effect of sample degradation can be reduced as far as possible and thus facilitates measurement of unstable species that are not amenable to analysis with conventional means.

Supplementary Note 2: Liouville Pathways Accessible by 125-Fold Phase Cycling

We list double-sided Feynman diagrams[2] corresponding to Liouville pathways that can be accessed by 1×5×5×5-fold phase cycling in Supplementary Figure 5. For the sake of simplicity, we only show one exemplary diagram per nonlinear signal contribution that leaves the system in an $|e\rangle\langle e|$ population state after the final laser-field interaction. For that purpose, we consider a simple four-level electronic system consisting of a ground state $|g\rangle$, a one-quantum state (1Q) $|e\rangle$, a two-quantum state (2Q) $|f\rangle$, and a three-quantum state (3Q) $|t\rangle$ (top left in Supplementary Figure 5). Each diagram is labeled at the top with the weighting parameters β, γ, and δ which are needed for its extraction from the raw data (via Eq. 1 of the main manuscript). Although all shown pathways exhibit a negative sign according to the Feynman diagram rules[2], the real part of all sixth-order signals is predominantly positive (with weak negative phase twists, see also Supplementary Figure 10). This is due to the additional factor i^2 from the perturbative expansion that must be multiplied with each sixth-order diagram, as noted in the main manuscript.

We now point out some benefits of particular signals. With 125-fold phase cycling, one is able to generate purely absorptive sixth-order 2Q-1Q (Figure 5 in the main manuscript) and 1Q-2Q 2D spectra (by adding the real parts of rephasing and nonrephasing contributions), where 2Q population dynamics as well as 0Q coherence between 2Q states can in principle be observed over T. Similarly as in the fourth-order regime, the sixth-order 1Q-2Q 2D spectrum may be less congested because the generation of 1Q coherences is restricted to superpositions between the ground and the first electronic excited state[3]. Sixth-order contributions may further probe 3Q coherences and how they couple to other states of the excited-state manifold by the sixth-order 1Q-3Q-1Q, 2Q-3Q-1Q, 1Q-3Q-2Q, and 2Q-3Q-2Q contributions. In the latter one, as well as in the sixth-order 2Q-1Q-2Q 3D spectrum, the bottom projection of the 3D solid yields the nonrephasing 2Q-2Q 2D correlation spectrum that may indicate coupling within the 2Q manifold by means of cross peaks.

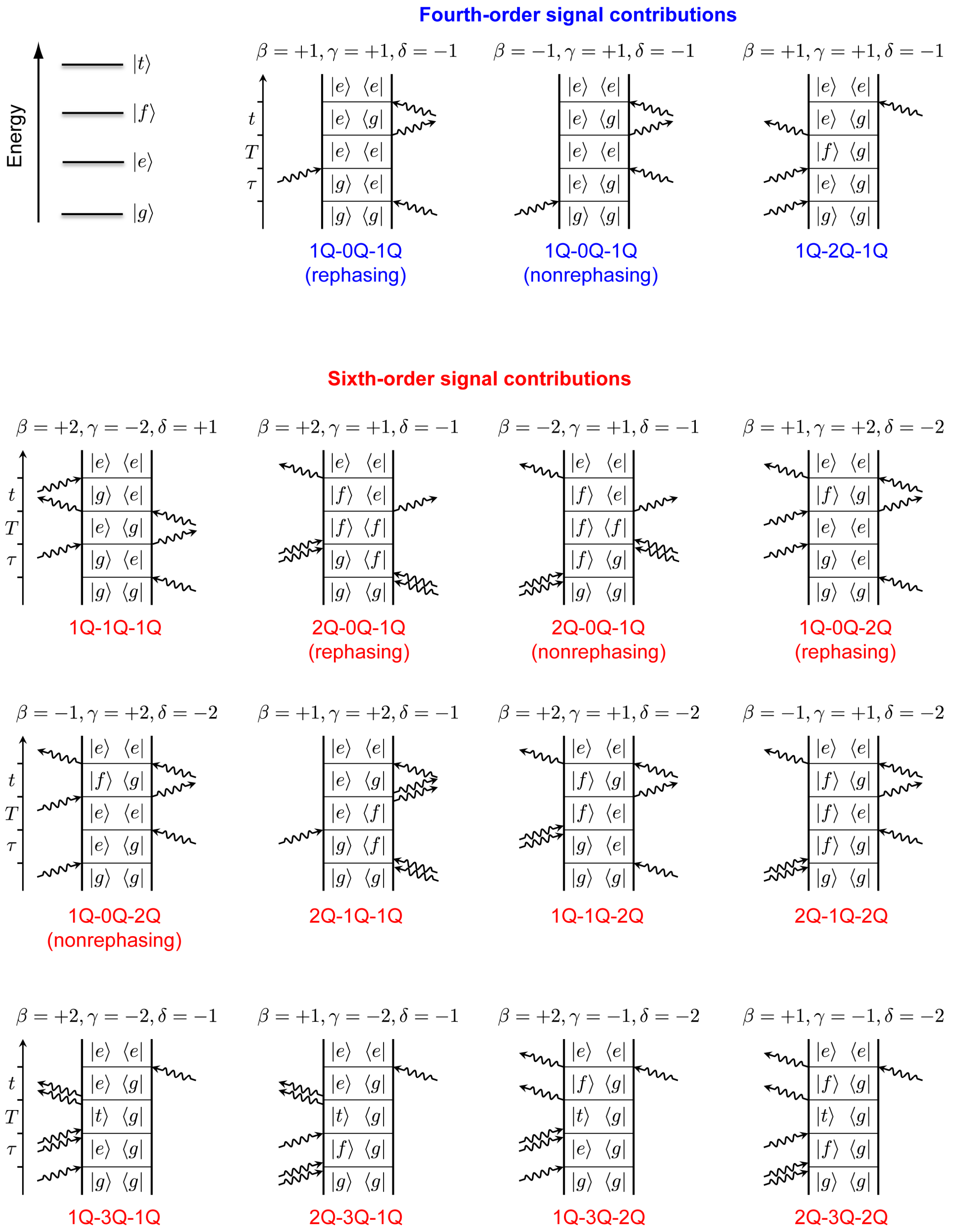

Supplementary Figure 5. Double-sided Feynman diagrams for all signal contributions that can be accessed via 125-fold phase cycling. On the basis of a four-level system (top left), one exemplary diagram for each fourth- and sixth-order nonlinear signal contribution is shown. The respective weighting factors β, γ, and δ are denoted on the top of each diagram.

Supplementary Note 3: Contamination of Fourth-Order Signals

A common fundamental assumption in third-order nonlinear spectroscopic experiments employing coherent detection is that the third-order signals dominate over the higher nonlinear orders (e.g., fifth-order signals). However, once fifth-order signals are present, these contaminate the third-order signals. This results from the emission of the signal of both nonlinear orders into the same phase matched direction, because one can in principle add zero to the wave vector of any of the incident beams (e.g., $0 = \mathbf{k}_1 - \mathbf{k}_1$). Due to the similar nature of phase matching and phase cycling, this effect is also expected in our experiment, meaning that for any pulse, interaction phases of, e.g., $0 = \varphi_1 - \varphi_1$ can be added. As a result, fourth-order signals can be contaminated by sixth-order signals, analogously to the case that third-order signals can be contaminated by fifth-order ones. We have discussed the latter previously in the context of our development of exciton–exciton-interaction 2D (EEI2D) spectroscopy[1]. In that work, we showed that the contamination of third-order signals, due to exciton–exciton annihilation, for example, can be characterized by separately and simultaneously measuring a fifth-order EEI2D signal.

In the present work, we compare the absolute-valued signal magnitudes of the fourth-order rephasing 1Q-0Q-1Q and sixth-order rephasing 2Q-0Q-1Q signals that we also simultaneously measure and find that the sixth-order signal has only 4.96% of the magnitude of the fourth-order one. Hence, because the sixth-order signal is over one magnitude weaker than the fourth-order one, any possible distortion would be at the 5% level or lower.

Considering all the evidence enables us to assign the nominally labeled “fourth-order spectra” indeed to result predominantly from the fourth-order nonlinear response.

Supplementary Note 4: Pathway Cancellation in the Fourth-Order Rephasing 1Q-0Q-1Q 3D Spectrum

The rephasing 1Q-0Q-1Q signal can in essence be described by depicting the response by double-sided Feynman diagrams which are shown in Supplementary Figure 6. Considering the phase signature of the signal, $\varphi_{\mathrm{R1Q0Q1Q}} = -\varphi_1+\varphi_2+\varphi_3-\varphi_4$, there are four pathway types Q_1, Q_2, Q_3, and Q_4 that can be constructed.

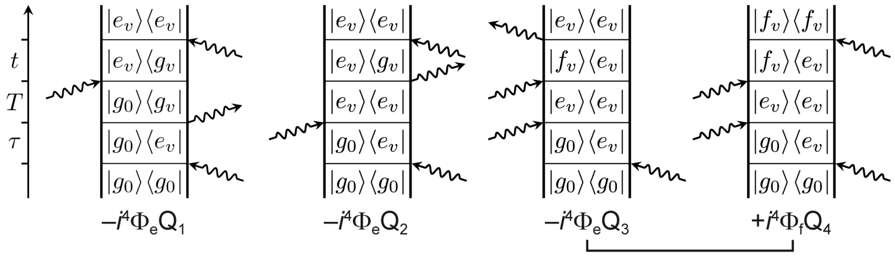

Supplementary Figure 6: Double-sided Feynman diagrams describing rephasing 1Q-0Q-1Q Liouville pathways. Each diagram is labeled according to its sign, where Φ denotes the pathway-specific fluorescence quantum yield that depends on the finally-prepared population state (subscript e or f).

Here, he subscript “*v*” in the bra’s and ket’s denotes the quantum of vibrational excitation, so that these pathway types can either be static (same vibrational quanta over the population time *T*) or oscillating (different sets of vibrational quanta over *T*). The pathway types Q_1 and Q_2, both having a negative sign, end in a $|e_v\rangle\langle e_v|$ population and contain the $|e_v\rangle\langle g_v|$ coherences that evolve over *t*. In contrast, pathway Q_3 and Q_4 probe the $|f_v\rangle\langle e_v|$ coherence dynamics over *t*. Hence, Q_3 and Q_4 would principally manifest as excited-state absorption (ESA) contributions along ω_t in a respective 3D spectrum. However, these two pathways are

opposite in sign. In the present case, fast internal conversion (IC) leads to identical weights Φ_e and Φ_f of pathways Q_3 and Q_4, respectively, because, if the system is left in a $|f_v\rangle\langle f_v|$ population, also one photon is emitted in the downward decay pathway $|f_v\rangle \rightarrow |e_v\rangle \rightarrow |g_v\rangle$. Thus, ESA contributions cannot be observed because of complete pathway cancellation. A similar situation is also evident regarding the 1Q-2Q-1Q pathways in Fig. 4b of the main manuscript. Consequently, pathways Q_1 and Q_2, to which the pathways in Fig. 3 of the manuscript also belong, represent the pathway types that entirely express the signal.

Supplementary Note 5: Experimental and Simulated Fourth-Order Nonrephasing 1Q-0Q-1Q 3D Spectra

We provide an exemplary detailed pathway analysis on the fourth-order nonrephasing 1Q-0Q-1Q 3D spectrum in Supplementary Figure 7. In principle, this kind of analysis can be conducted for every signal contribution that can be extracted from the raw dataset. Such a full analysis of all signal contributions is, however, beyond the scope of this work. We extract the nonrephasing 1Q-0Q-1Q signal contribution from the raw data by employing weights corresponding to its phase signature[3] of $\varphi_{NR1Q0Q1Q} = \varphi_1 - \varphi_2 + \varphi_3 - \varphi_4$. In order to discuss its peak positions, we choose the absolute-valued representation. The experimental and simulated 3D spectra are displayed in Supplementary Figure 7a and 7b, respectively, whereas corresponding double-sided Feynman diagrams are shown in Supplementary Figure 7c. We do not consider diagrams involving the higher excited state because every negatively signed excited-state absorption (ESA) pathway has a positively signed analogue[4] which leads to destructive interference of these pathways due to the high internal conversion rate as employed in the model. A similar situation is evident in the rephasing 1Q-0Q-1Q spectra (see also Supplementary Note 4). In general, the 1Q-0Q-1Q pathways can further be classified as either “static” or “beating”, depending on whether the dynamic evolution of a population density matrix element (highlighted in green) or a 0Q coherence density matrix element (red and blue, corresponding to positive and negative frequency shift, respectively) is probed over T.

Overall, the nonrephasing 1Q-0Q-1Q 3D spectrum shows a similar peak arrangement as its rephasing analogue (Figure 3 in the main manuscript). It features two diagonal (A, D) and two cross peaks (B, C) in the $\Delta\hbar\omega_T = 0$ plane. In contrast to the positively and negatively shifted 0Q peaks in the rephasing 1Q-0Q-1Q 3D spectrum of the main manuscript (Figure 3), in the nonrephasing 1Q-0Q-1Q 3D spectrum, a peak E that is shifted by +0.17 eV on the 0Q axis appears above the diagonal peak D in the ($\hbar\omega_\tau$, $\Delta\hbar\omega_T$, $\hbar\omega_t$) plane. However, it is so weak in magnitude that it can only be visualized if we mask out the strong feature A at lower 1Q energies. Thus, we isolate the voxels within the spectral volume that spans the region in the vicinity of peak D (Supplementary Figure 7a and 7b, right) to make peak E visible. The latter reflects a superposition of two pathways that probe 0Q coherence both in S_0 and S_1 (see diagrams Q_{13} and Q_{14} in Supplementary Figure 7c). The amplitude of this peak is so low because the energies of the involved electronic transitions are only poorly covered by the employed laser spectrum. Diagram Q_{10} corresponds to an additional 3D peak which is expected to appear at a coordinate of ($\hbar\omega_\tau$, $\hbar\omega_T$, $\hbar\omega_t$) = (2.06, −0.17, 2.06) eV, where the negative 0Q coherence shift corresponds to excited-state vibrational coherence. However, it is masked by the lineshape of the dominating peak A.

Spectral filtering was shown to be a useful method to eliminate certain Liouville pathways[5]. In our experiments, we did not utilize it on purpose. However, because the laser spectrum is blue-shifted from the main absorption band, some pathways are also eliminated. This means, because the laser spectrum does not cover the energy of the transition between $|e_0\rangle$ and $|g_1\rangle$ (1.89 eV), pathways that involve this transition do not contribute to the signal. Apparently, this mostly affects beating pathways. We indicate this by red crosses in Supplementary Figure 7c.

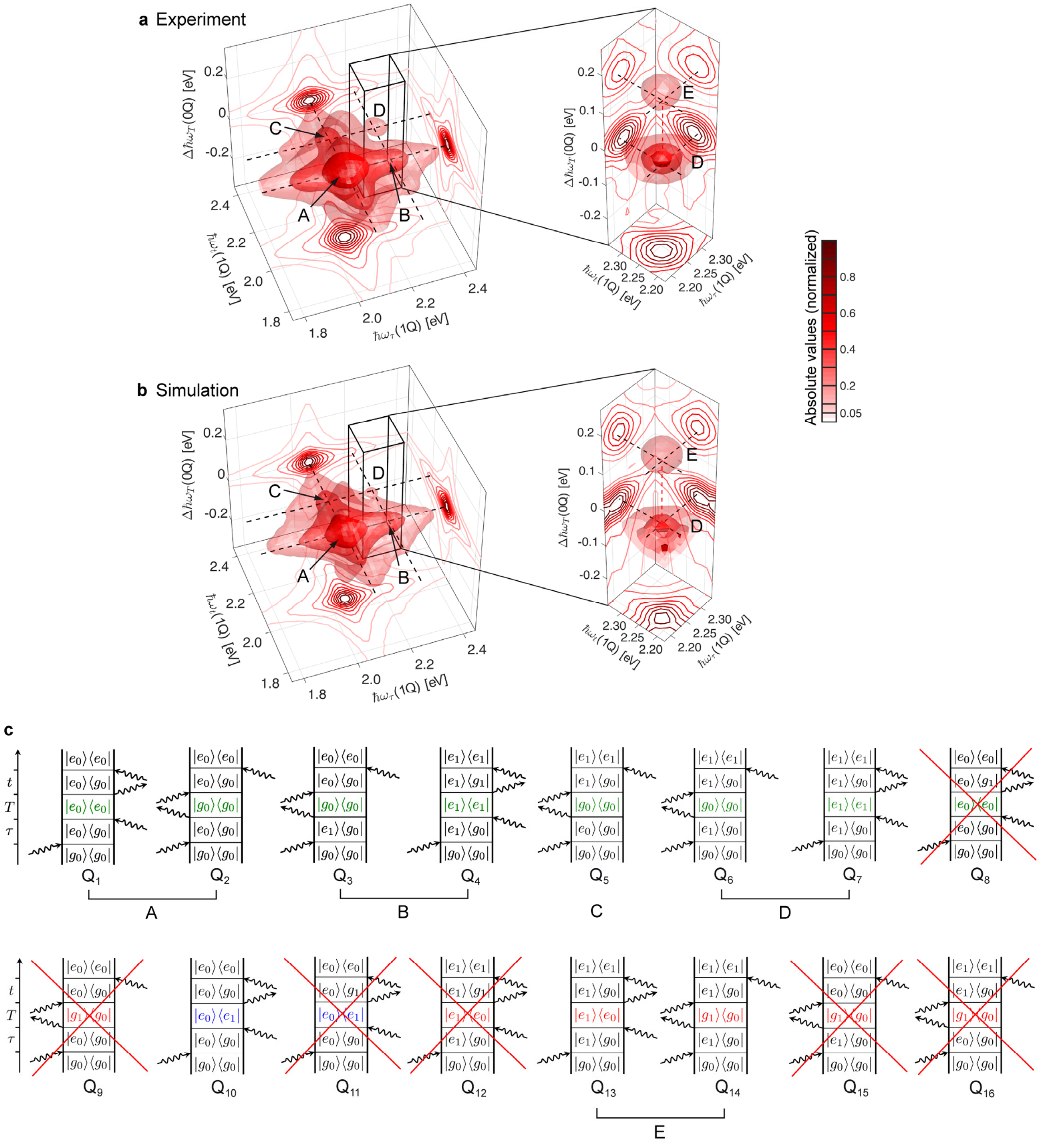

Supplementary Figure 7. Fourth-order nonrephasing 1Q-0Q-1Q 3D spectrum of TIPS-TAP^{2-} in THF. **a** Experimental 3D spectrum shown along with **b** the simulated 3D spectrum. Within both experimental and simulated 3D spectra, the energies of the levels $|e_0\rangle$ (2.06 eV) and $|e_1\rangle$ (2.23 eV) are indicated by dashed black lines. Isosurfaces are drawn at 3.9, 12, and 35% of the maximal signal amplitude. Contour lines of the 2D projections of the spectral solids are drawn at linearly spaced levels of the normalized signal amplitude with an additional contour line at 0.05. The spectral volume inside the black cuboid, which is centered around peak D, is isolated and shown on the right. There, isosurfaces are drawn at 40, 70, and 90% of the isolated maximal signal amplitude and the red dashed line denotes an energy shift of +0.17 eV. **c** Possible double-sided Feynman diagrams that contribute to the signal. Non-oscillatory density matrix elements are highlighted in green whereas oscillatory 0Q coherence is highlighted in red and blue, according to positive and negative oscillation frequency, respectively. Certain pathways are grouped under the letters A to E in order to label the respective features observed in the 3D spectra. Non-contributing pathways, which involve a transition between $|e_0\rangle$ and $|g_1\rangle$, are crossed out.

Supplementary Note 6: Description of the Simulation Model

For simulations, we solve the Lindblad master equation[6], which can be written in a superoperator form,

$$\frac{\partial}{\partial t'}\rho(t') = -i\mathcal{L}_0\rho(t') - \mathcal{L}_{\mathrm{SO}}\rho(t'). \tag{1}$$

In terms of the density matrix $\rho(t')$, the first term on the right side of Supplementary Eq. 1 is

$$\mathcal{L}_0\,\rho(t') = \frac{1}{\hbar}[H(t'),\rho(t')], \tag{2}$$

and the second term reads

$$\mathcal{L}_{\mathrm{SO}}\rho(t') = \sum_j \frac{1}{T_j}\mathcal{L}_j\rho(t')\mathcal{L}_j^\dagger - \frac{1}{2}\mathcal{L}_j^\dagger\mathcal{L}_j\rho(t') - \frac{1}{2}\rho(t')\mathcal{L}_j^\dagger\mathcal{L}_j, \tag{3}$$

with T_j as the time being associated with a dissipation process that induces decoherence by pure dephasing and population relaxation. The Lindblad operators $\mathcal{L}_j$ are defined as $\mathcal{L}_j = a_n^\dagger a_n$ for pure dephasing and $\mathcal{L}_j = a_n^\dagger a_m$ with $n \neq m$ for a population relaxation process, where $a^\dagger$ and a denote the creation and annihilation operators, respectively[7,8]. The superoperator formulation leads to a reduction of computational cost of calculations with a large amount of external light fields, which is 421875 in the present case.

The Hamiltonian $H(t')$ in Supplementary Eq. 2 is given by

$$H(t') = H_0 + H_I(t'), \tag{4}$$

where the time-independent Hamiltonian H_0 is

$$H_0 = \hbar\omega_n \sum_n^N |n\rangle\langle n| \tag{5}$$

with energy eigenstates $|n\rangle$ and associated frequencies ω_n. The interaction Hamiltonian $H_I(t')$ is

$$H_I(t') = \gamma_{\mathrm{ext}}E(t')\sum_{n\neq m}\mu_{nm}(|n\rangle\langle m| + |m\rangle\langle n|), \tag{6}$$

with the external field coupling strength $\gamma_{\mathrm{ext}} = 0.3\times10^{-3}$ and transition dipole moments μ_{nm} between states $|n\rangle$ and $|m\rangle$. In Supplementary Eq. 6, the external light fields $E(t')$ are four-pulse sequences with pulse-specific phases φ_k and defined as

$$\begin{aligned} E(t') = {} & \exp\left(-\frac{4\ln 2}{{\tau_p}^2}(t'-t_0)^2\right)\exp(i\omega_0(t'-\gamma_0 t_0) - i\varphi_1) \\ & + \exp\left(-\frac{4\ln 2}{{\tau_p}^2}(t'-t_0+\tau)^2\right)\exp(i\omega_0(t'-\gamma_0(t_0+\tau)) - i\varphi_2) \\ & + \exp\left(-\frac{4\ln 2}{{\tau_p}^2}(t'-t_0+\tau+T)^2\right)\exp(i\omega_0(t'-\gamma_0(t_0+\tau+T)) - i\varphi_3) \\ & + \exp\left(-\frac{4\ln 2}{{\tau_p}^2}(t'-t_0+\tau+T+t)^2\right)\exp(i\omega_0(t'-\gamma_0(t_0+\tau+T+t)) - i\varphi_4), \end{aligned} \tag{7}$$

where t_0 is an offset (set to 100 fs), $\tau, T,$ and t are the interpulse delays, which are sampled identical to the experimental parameters, ω_0 is the central frequency (3.19 rad/fs, corresponding to 2.10 eV), and τ_p is the pulse duration given by the intensity full width at half maximum, which is set to 17 fs. The parameter γ_0 is set to zero (fully rotating frame), allowing to take larger sampling steps like in the experiment. We employ a six-level energy level scheme, representing a model with three electronic states, where each state is coupled to a single vibrational mode with an energy of 0.17 eV, assuming equal vibrational frequencies in all electronic states (see Figure 2b in the main manuscript). The model neglects solvent and dynamic Stokes shift effects as well as possible singlet fission mechanisms. It was previously reported that TIPS-pentacene

exhibits singlet fission in highly concentrated solutions[9]; however, even if we assume that such a process could be present in the dianionic tetraaza-compound, it is improbable regarding the comparably low concentration regime (0.4 mM) we used in our experiment.

For calculating the transition dipole moments, we estimate the Huang-Rhys factor S_{HR} of the 0.17 eV mode that is coupled to the $S_0 \rightarrow S_1$ transition to be 0.6 from linear absorption data. The transition dipole moments μ_{nm} between electronic level pairs $|a_n\rangle$ and $|b_m\rangle$ with vibrational quanta n and m are thereby defined as[10]

$$\mu_{b_0 a_0} = \mu \exp(-S_{\mathrm{HR}}/2), \tag{8}$$

$$\mu_{b_1 a_0} = \mu_{b_0 a_1} = \sqrt{S_{\mathrm{HR}}}\mu \exp(-S_{\mathrm{HR}}/2), \tag{9}$$

$$\mu_{b_1 a_1} = (1 - S_{\mathrm{HR}})\mu \exp(-S_{\mathrm{HR}}/2), \tag{10}$$

with an "overall" transition dipole moment μ. In order to estimate S_{HR} of the same mode coupled to the $S_1 \rightarrow S_{74}$ transition, we carry out a screening of S_{HR} until best agreement between the experimental and the simulated 1Q-2Q-1Q 3D spectrum is achieved, where we vary S_{HR} from 0.6 to 0.05 in steps of 0.05. A S_{HR} of 0.05 reproduces the 3D solid best, especially the amplitude of the shoulder above 4.10 eV along the 2Q axis (see Figure 4 in the main manuscript). Further, we scale the strengths of the $|e_n\rangle \rightarrow |f_n\rangle$ transitions relative to the $|g_n\rangle \rightarrow |e_n\rangle$ transitions according to the ratio of oscillator strengths f as received from the TD-DFT calculation (see Supplementary Note 7),

$$\frac{f_{S_0 \rightarrow S_1}}{f_{S_1 \rightarrow S_{74}}} = \frac{|\mu_{eg}|^2}{|\mu_{fe}|^2}, \tag{11}$$

which leads to $\mu_{fe} = 0.64\ \mu_{eg}$. The resulting transition moments are summarized in Supplementary Table 1.

We employ a ladder-type population relaxation down to the global ground state $|g_0\rangle$, where we set the relaxation constant of $|e_0\rangle$ to lower states to 6.1 ns according to the experimentally determined fluorescence lifetime of the molecule[11]. For internal conversion, we assume a 100 fs relaxation time where we further estimate a 6 ps relaxation time for vibrationally excited populations in each electronic state.

The general procedure for estimating the pure dephasing times is as follows. First, a minimal four-level system with the levels $|g_0\rangle$, $|g_1\rangle$, $|e_0\rangle$, and $|e_1\rangle$ is considered and the rephasing and nonrephasing 1Q-0Q-1Q signals are simulated and compared to the experimental result. A manual adjustment of the pure 1Q dephasing time in steps of 10 fs is then performed until best agreement with experimental data. This value is also used as a starting value for the dephasing time of coherences between the $|e_n\rangle$ and $|f_n\rangle$ as well as the $|g_n\rangle$ and $|f_n\rangle$ manifold. Then, a comparison between experimental and simulated 1Q-2Q-1Q 3D spectra is used as an additional feedback for the iterative manual refinement of the $|f_n\rangle\langle e_n|$ and $|f_n\rangle\langle g_n|$ dephasing times, leading to the parameters given in Supplementary Table 1. All calculations were conducted on a CPU cluster with 72 cores and 1 TB RAM using the parallel computing toolbox in MatLab R2018a, where the calculation for each pulse sequence is conducted separately on a single core, so that a full simulation dataset is generated in approximately 2 hours.

Supplementary Table 1. Simulation parameters. Table showing transition moments and pure dephasing times between the states employed in the model.

Transition moments					
$S_0 \rightarrow S_1$		$S_1 \rightarrow S_{74}$		$S_0 \rightarrow S_{74}$	
$\mu_{e_0 g_0}$	0.74	$\mu_{f_0 e_0}$	0.62	$\mu_{f_0 g_0}$	0
$\mu_{e_1 g_0}$	0.57	$\mu_{f_1 e_0}$	0.14	$\mu_{f_1 g_0}$	0
$\mu_{e_0 g_1}$	0.57	$\mu_{f_0 e_1}$	0.14	$\mu_{f_0 g_1}$	0
$\mu_{e_1 g_1}$	0.27	$\mu_{f_1 e_1}$	0.59	$\mu_{f_1 g_1}$	0
Pure dephasing time constants T_2^*					
$\|e_n\rangle\langle g_n\|$	90 fs	$\|f_n\rangle\langle e_n\|$	300 fs	$\|f_n\rangle\langle g_n\|$	100 fs

Supplementary Note 7: Quantum-Chemical Calculations

In order to evaluate the origin of the two-quantum (2Q) resonance at 4.10 eV in the 2Q-associated 3D spectra, we performed quantum chemical calculations by using density functional theory (DFT) and time-dependent density functional theory (TD-DFT). All calculations were carried out with the Gaussian program package[12]. The ground state equilibrium structure of TIPS-TAP^{2-} was optimized with DFT/CAM-B3LYP/6-311G** and employing a polarizable continuum solvent model for tetrahydrofuran (THF) solvation. TD-DFT calculations were performed on the optimized structure with BLYP functional and 6-31+G* basis set for the first 100 vertical transitions ($S_0 \rightarrow S_n$). Subsequently, $S_1 \rightarrow S_n$ transitions were obtained by computing transition dipole moments between excited states. In the $S_0 \rightarrow S_n$ spectrum (Supplementary Figure 8), the $S_0 \rightarrow S_1$ transition energy (590 nm, 2.10 eV) is in good agreement with the experimentally observed peak (602 nm, 2.06 eV). There are some higher excited states with nonzero oscillator strength. However, at nearly twice the energy of the $S_0 \rightarrow S_1$ transition (295 nm, 4.20 eV) there is no transition with significant oscillator strength present in the calculation which is analogous to experimental observations. Thus, this reflects that the experimentally observed 2Q state at 4.10 eV is unlikely to be excited via a single one-photon transition due to inversion symmetry of the molecule.

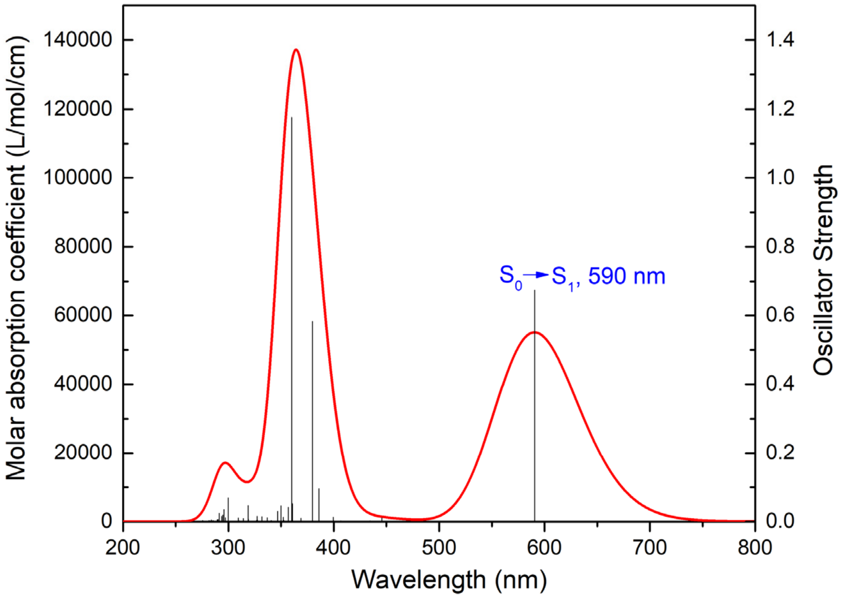

Supplementary Figure 8. Calculated $S_0 \rightarrow S_n$ (n = 1, …, 100) absorption spectrum of TIPS-TAP^{2-}. The spectrum is broadened using Gaussian functions with a full width at half maximum of 0.33 eV.

In contrast, the $S_1 \rightarrow S_n$ absorption spectrum (Supplementary Figure 9) features a transition into S_{74} (579 nm, 2.14 eV) with relatively high oscillator strength at nearly the same energy as the $S_0 \rightarrow S_1$ transition. There is also a second optically bright transition at 1157 nm (1.07 eV) which is not of relevance within our experiments because it is not covered by the employed laser spectrum. Hence, we conclude that the 2Q resonance at 4.10 eV in the 3D spectra origins from S_{74} which is only accessible via two consecutive one-photon transitions $S_0 \rightarrow S_1 \rightarrow S_{74}$. The calculated oscillator strength of the direct $S_0 \rightarrow S_{74}$ one-photon transition is zero which further underlines that S_{74} is a two-photon allowed but a one-photon forbidden electronic state.

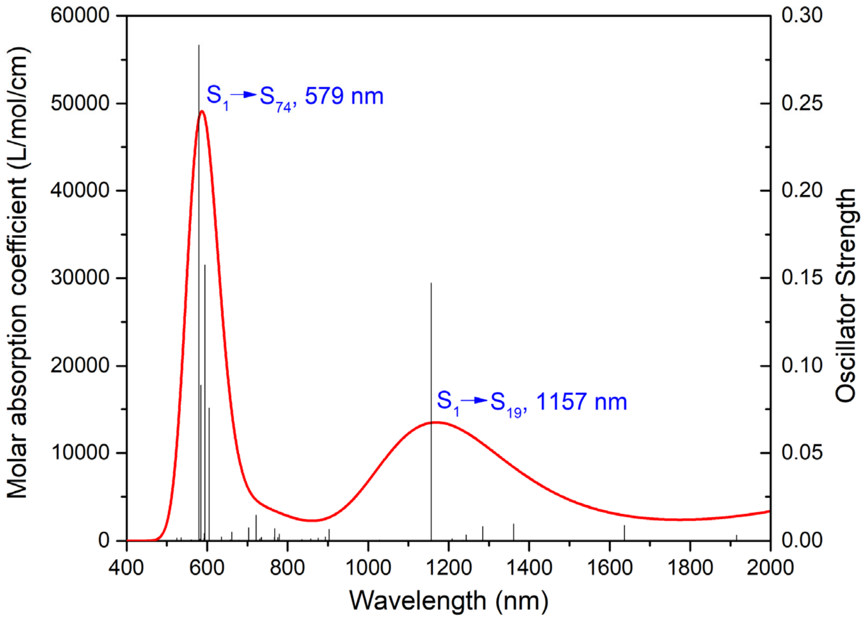

Supplementary Figure 9. Calculated $S_1 \rightarrow S_n$ (n = 2, …, 100) absorption spectrum of TIPS-TAP^{2-}. The spectrum is broadened using Gaussian functions with a full width at half maximum of 0.33 eV.

Supplementary Note 8: Experimental and Simulated Rephasing Fourth- and Sixth-Order 2D Spectra

Supplementary Figure 10 shows a comparison between experimental (top row) and simulated (bottom row) rephasing 1Q-1Q (left column) and rephasing 2Q-1Q (right column) 2D spectra at T = 18 fs. While the rephasing 1Q-1Q spectrum has a predominant negative signal, which corresponds to a fourth-order response, the rephasing 2Q-1Q spectrum is opposite in sign because it stems from a sixth-order response. Both lineshapes are phase-twisted and elongated along the diagonal line as typical for rephasing signals. While in the fourth-order process, 1Q coherences are rephased to each other, in the sixth-order process the 2Q coherence is rephased after conversion into a 1Q coherence, thus both the 1Q and 2Q inhomogeneity are convoluted into the response[13]. We observe that the antidiagonal linewidth is similar in both spectra, indicating that the 2Q states do not contribute any substantial inhomogeneity in addition to the pure 1Q inhomogeneity. When further comparing the spectra, the largely different Huang-Rhys factors between different electronic states (see also Supplementary Note 6) is reflected by means of different peak amplitudes of the sidepeaks in the 1Q and 2Q domain. These cross peaks correspond to coupling to vibrationally excited states. The cross peak at ($\hbar\omega_\tau$, $\hbar\omega_t$) = (2.06, 2.23) eV in the fourth-order signal is much lower in amplitude than the cross peak at (4.27, 2.23) eV. At first glance, this seems contradictory though, however, one must take into account that the laser spectrum has its maximum amplitude at 2.10 eV (see Figure 2a in the main manuscript). Thus, in the two-photon domain, 2Q features in the vicinity of twice that energy will be enhanced compared to 2Q resonances at lower energies. This is further increased because of an overall six-fold multiplication of the laser spectrum into the signal.

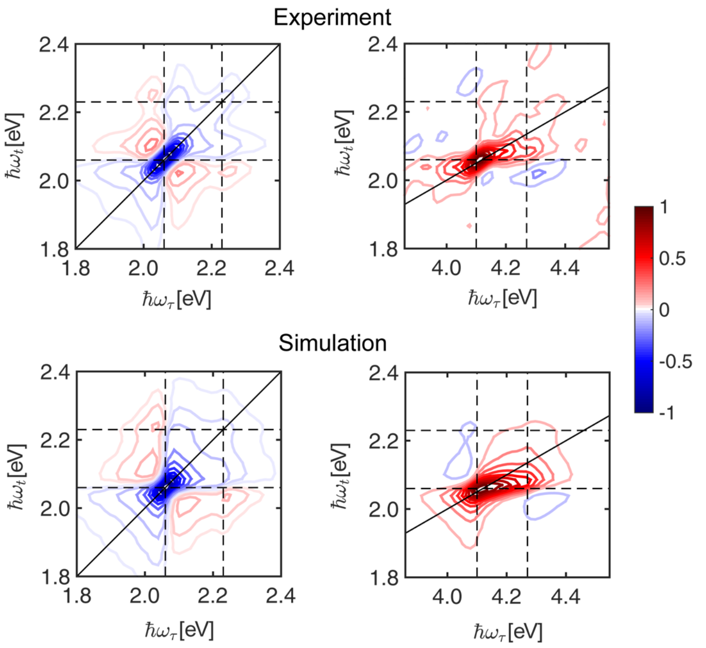

Supplementary Figure 10. Rephasing fourth- and sixth-order 2D spectra of TIPS-TAP^{2-} in THF. Experimental (top) and simulated (bottom) real-valued fourth-order rephasing 1Q-1Q (left column) and sixth-order rephasing 2Q-1Q (right column) 2D spectra, shown at $T = 18$ fs each. All spectra are normalized to their respective maximal absolute value. Contour lines are drawn at identical values in both experimental and simulated spectra. Black dashed lines denote the energies of the $|e_\nu\rangle$ and $|f_\nu\rangle$ states that are considered in the model.

Supplementary Note 9: Many-Particle Excitations

In a recent communication, it was shown that to achieve many-particle signal detection of non-interacting particles, phase-sensitive detection such as phase cycling, phase modulation or photon coincidence detection can be utilized[14]. In this respect, the detection of fluorescence of N non-interacting particles, excited by a pair of pulses with mutual phase $\varphi_{21} = \varphi_1 - \varphi_2$ and separated by delay T, is considered. By extracting, for instance, from the detected fluorescence the part oscillating at $\pm 2\varphi_{21}$ over T, the discussion can be restricted to the observation of two-particle operators. Starting from a single particle with a ground state $|g\rangle$ and an excited state $|e\rangle$, we can thus formulate a two-particle system in a collective basis with the resulting energy-level scheme depicted in Supplementary Figure 11a. In the language of Liouville-space pathways, the contributions oscillating at $\pm 2\varphi_{21}$ correspond to the pathways where both particles j and k are in the optical coherence during T, which is expressed as the two-quantum coherence $|e_j e_k\rangle\langle g_j g_k|$ in Supplementary Figures 11b-e. Within the two-particle basis, the two-particle projection operators can be expressed as:

$$A_{jk}^{ee} = |e_j\rangle\langle e_j| \otimes |e_k\rangle\langle e_k|, \tag{12}$$

$$A_{jk}^{eg} = |e_j\rangle\langle e_j| \otimes |g_k\rangle\langle g_k|, \tag{13}$$

$$A_{jk}^{ge} = |g_j\rangle\langle g_j| \otimes |e_k\rangle\langle e_k|, \tag{14}$$

$$A_{jk}^{gg} = |g_j\rangle\langle g_j| \otimes |g_k\rangle\langle g_k|, \tag{15}$$

where the resulting double-sided Feynman diagrams are shown in Supplementary Figures 11b-e, respectively. In Ref. 14, it is argued that the only operator observed is A_{jk}^{ee} which corresponds to pathway Q_{jk}^{ee} in Supplementary Figure 11b. In that case, both particles are in their excited state which results in a

weight of two because of the emission of two photons by fluorescence. We believe that it depends on the type of detection which two-particle operators are observed. While in the case of two-photon coincidence detection, contributions from only Q_{jk}^{ee} could indeed be observed. In the case of conventional, non-coincidence, fluorescence detection as in the present work, we also detect one-photon contributions, that is, A_{jk}^{ee}, A_{jk}^{eg}, and A_{jk}^{ge} all contribute. Depicting the respective parts of the response by Liouville-space pathways, we find that while Q_{jk}^{ee} contributes with +2, Q_{jk}^{eg} and Q_{jk}^{ge} contribute each by −1 (Q_{jk}^{gg} does not contribute at all because A_{jk}^{gg} projects to the ground state of both particles which does not generate any fluorescence). As a result, the pathways cancel each other, leaving no contribution from the multi-particle states. In Ref. 14, this issue is addressed in the discussion in the last paragraph, arguing that the two-particle contribution does not affect the one-particle fluorescence. Our argumentation is not in contradiction with this statement; we rather argue that one has to consider all observable two-particle operators and not select just one of them.

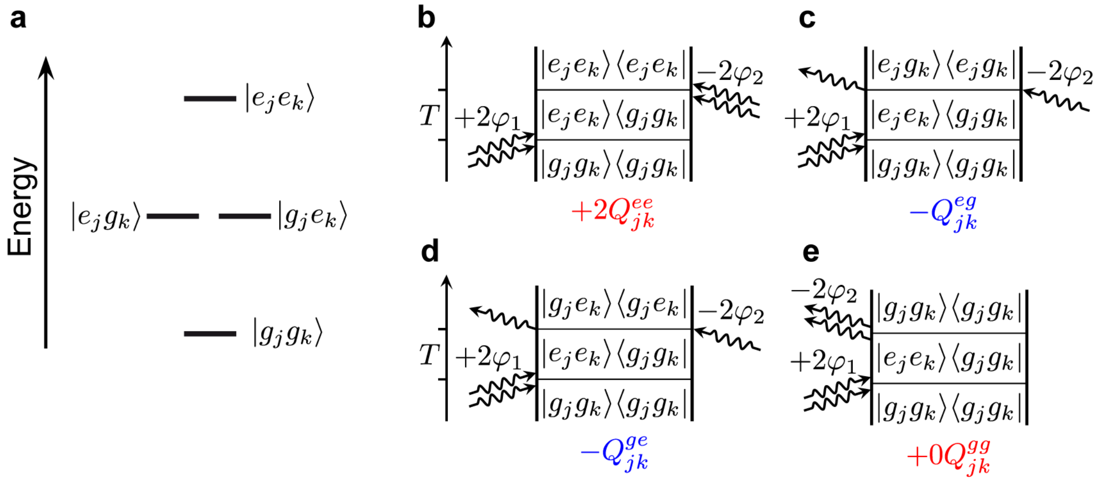

Supplemetary Figure 11. Many-particle excitation pathways. **a** Energy-level scheme of two non-interacting two-level systems in a collective basis. **b-e** Double-sided Feynman diagrams that can be accessed by extracting a signal contribution oscillating with $+2\varphi_{21}$ over T by a two-pulse experiment with fluorescence detection. Here, pathways that result from all possible two-particle projection operators are drawn, where the respective fluorescence yield is indicated by a prefactor denoted at the bottom of each diagram.

The assertions made above can directly be transferred to the case of our sixth-order signals where the respective diagrams would display extended versions in terms of additional delays and pulses of those depicted in Supplementary Figure 11b-d. Formulating, e.g., the sixth-order rephasing 2Q-0Q-1Q pathways by employing a collective basis and the two-particle projector A_{jk}^{ee}, the sign of the resulting pathway would be negative (that is, because of four interactions from the right in the corresponding double-sided Feynman diagrams, giving an initially positive sign which is then multiplied by the prefactor of $i^6 = -1$ from the perturbative expansion). In our experiment, however, we observe a positive phase in the real part of the sixth-order signals which can only be explained by considering single-particle excitations, as it is displayed by the double-sided Feynman diagram of Figure 6d of the main manuscript. Moreover, for our simulations we explicitly formulate our model in a single-particle basis and use parameters from TD-DFT calculations that confirmed the presence of a single-particle doubly excited state $|f\rangle$. Regarding the excellent agreement with the simulation (see manuscript Fig. 5), we conclude that the single-particle model is fully valid.

In conclusion, many-particle excitations do not contribute to the signals we acquired in our experiment because of complete cancellation of pathways corresponding to the complete manifold of two-particle projection operators. Thus, our signals must stem from microscopic quantum coherence of individual molecules that can be described in a single-particle basis as it is treated in the main manuscript.

Supplementary Note 10: Cancelling Sixth-Order Rephasing 2Q-0Q-1Q Pathways

We show double-sided Feynman diagrams of the rephasing 2Q-0Q-1Q process that probe 0Q coherence within the 2Q state over T in Supplementary Figure 12. It is evident that each positive diagram has an oppositely signed pendant. These two diagrams only differ in their final population state. As noted in the main manuscript, internal conversion from the 2Q-excited states to the 1Q-excited states leads to $\Phi_e = \Phi_f$, meaning that each diagram contributes with equal magnitude to the signal. Thus, both the dynamic evolution of the $|f_1\rangle\langle f_0|$ (highlighted in red) and $|f_0\rangle\langle f_1|$ (highlighted in blue) coherences cannot be probed because the underlying pathways cancel in pairs. This is also valid for diagrams that contain the population density matrix elements $|f_0\rangle\langle f_0|$ and $|f_1\rangle\langle f_1|$ over T (not shown).

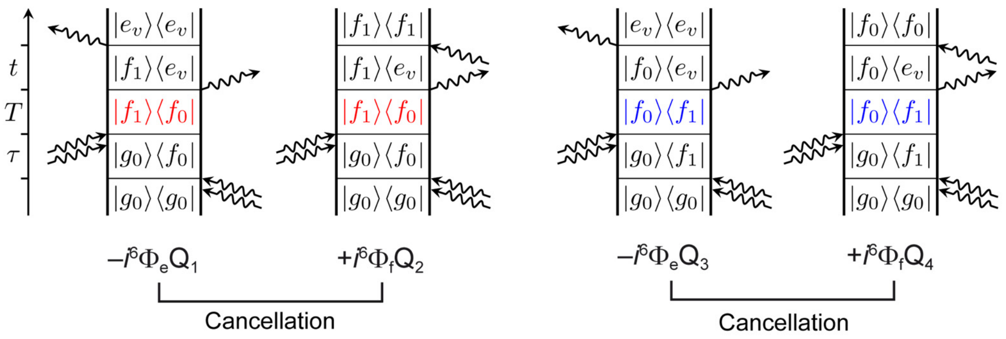

Supplementary Figure 12. Cancelling sixth-order diagrams. Double-sided Feynman diagrams are displayed of the sixth-order rephasing 2Q-0Q-1Q process that include 0Q coherence between vibrational levels of the 2Q state (highlighting in red and blue denotes positive and negative oscillation frequency, respectively). Due to internal conversion, the pathway-specific quantum yields are $\Phi_e = \Phi_f$, so that the diagrams Q_1 and Q_2 as well as Q_3 and Q_4 cancel in pairs.

Supplementary Note 11: Simulated Fourth- and Sixth-Order 0Q Transients

In order to evaluate the phase-shifted 0Q coherence dynamics from fourth-order rephasing 1Q-0Q-1Q and sixth-order rephasing 2Q-0Q-1Q contributions in Figure 6 of the main manuscript, we show the respective result from a simulation in Supplementary Figure 13, where we employed 43 steps along T in order to receive higher-resolved transients. The transients were taken at above-diagonal cross-peak positions in the 1Q-1Q and 2Q-1Q 2D projections of the 3D contributions, which is identical to those positions taken in the experimental ones. The same oscillation period (≈ 24 fs) and the π phase shift between the transients of different orders are in excellent agreement with experimental observations.

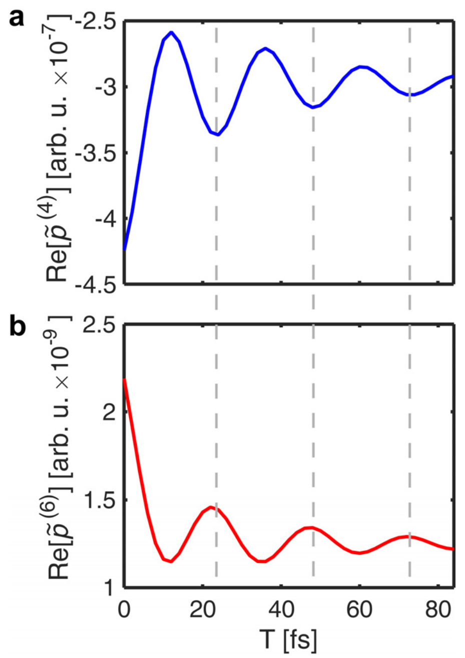

Supplementary Figure 13. Simulated fourth- and sixth-order 0Q coherence dynamics of TIPS-TAP^{2-}. The real-valued 0Q coherence signatures over T which are received from **a** the simulated fourth-order rephasing 1Q-0Q-1Q ($\tilde{p}^{(4)}$) and **b** sixth-order rephasing 2Q-0Q-1Q ($\tilde{p}^{(6)}$) contributions show a π phase shift.

Supplementary References

1. Dostál, J. et al. Direct observation of exciton–exciton interactions. *Nat. Commun.* **9**, 2466 (2018).
2. Mukamel, S. *Principles of nonlinear optical spectroscopy* (Oxford University Press, Oxford, 1995).
3. Tan, H.-S. Theory and phase-cycling scheme selection principles of collinear phase coherent multi-dimensional optical spectroscopy. *J. Chem. Phys.* **129**, 124501 (2008).
4. Perdomo-Ortiz, A., Widom, J. R., Lott, G. A., Aspuru-Guzik, A. & Marcus, A. H. Conformation and electronic population transfer in membrane-supported self-assembled porphyrin dimers by 2D fluorescence spectroscopy. *J. Phys. Chem. B* **116**, 10757–10770 (2012).
5. Green, D., V. A. Camargo, F., Heisler, I. A., Dijkstra, A. G. & Jones, G. A. Spectral filtering as a tool for two-dimensional spectroscopy: A theoretical model. *J. Phys. Chem. A* **122**, 6206–6213 (2018).
6. Lindblad, G. On the generators of quantum dynamical semigroups. *Commun. Math. Phys.* **48**, 119–130 (1976).
7. Vella, E. et al. Ultrafast decoherence dynamics govern photocarrier generation efficiencies in polymer solar cells. *Sci. Rep.* **6**, 29437 (2016).

8. Damtie, F. A., Wacker, A., Pullerits, T. & Karki, K. J. Two-dimensional action spectroscopy of excitonic systems: Explicit simulation using a phase-modulation technique. *Phys. Rev. A* **96**, 053830 (2017).
9. Walker, B. J., Musser, A. J., Beljonne, D. & Friend, R. H. Singlet exciton fission in solution. *Nat. Chem.* **5**, 1019–1024 (2013).
10. van Amerongen, H., Valkunas, L. & van Grondelle, R. *Photosynthetic Excitons* (World Scientific Publishing Co. Pte. Ltd., 2000).
11. Ji, L. et al. Preparation, properties, and structures of the radical anions and dianions of azapentacenes. *J. Am. Chem. Soc.* **139**, 15968–15976 (2017).
12. Frisch, M. J. et al. Gaussian 09, Revision D.01; Gaussian, Inc., Wallingford CT, 2009.
13. Turner, D. B., Stone, K. W., Gundogdu, K. & Nelson, K. A. Invited Article: The coherent optical laser beam recombination technique (COLBERT) spectrometer: Coherent multidimensional spectroscopy made easier. *Rev. Sci. Instrum.* **82**, 081301 (2011).
14. Mukamel, S. Communication: The origin of many-particle signals in nonlinear optical spectroscopy of non-interacting particles. *J. Chem. Phys.* **145**, 041102 (2016).
